# Satellite-aided survey sampling and implementation in low- and middle-income contexts: a low-cost/low-tech alternative

**DOI:** 10.1186/s12982-015-0041-8

**Published:** 2015-12-23

**Authors:** Marco J. Haenssgen

**Affiliations:** Centre for Tropical Medicine and Global Health, Nuffield Department of Medicine, University of Oxford, Oxford, UK; CABDyN Complexity Centre, Saïd Business School, University of Oxford, Oxford, UK; Green Templeton College, University of Oxford, Oxford, UK

**Keywords:** Survey, Sampling, Implementation, Rural, India, China, Google Maps, Bing Maps

## Abstract

**Background:**

The increasing availability of online maps, satellite imagery, and digital technology can ease common constraints of survey sampling in low- and middle-income countries. However, existing approaches require specialised software and user skills, professional GPS equipment, and/or commercial data sources; they tend to neglect spatial sampling considerations when using satellite maps; and they continue to face implementation challenges analogous to conventional survey implementation methods. This paper presents an alternative way of utilising satellite maps and digital aides that aims to address these challenges.

**Results:**

The case studies of two rural household surveys in Rajasthan (India) and Gansu (China) compare conventional survey sampling and implementation techniques with the use of online map services such as Google, Bing, and HERE maps. Modern yet basic digital technology can be integrated into the processes of preparing, implementing, and monitoring a rural household survey. Satellite-aided systematic random sampling enhanced the spatial representativeness of the village samples and entailed savings of approximately £4000 compared to conventional household listing, while reducing the duration of the main survey by at least 25 %.

**Conclusion:**

This low-cost/low-tech satellite-aided survey sampling approach can be useful for student researchers and resource-constrained research projects operating in low- and middle-income contexts with high survey implementation costs. While achieving transparent and efficient survey implementation at low costs, researchers aiming to adopt a similar process should be aware of the locational, technical, and logistical requirements as well as the methodological challenges of this strategy.

## Background

Survey research in low- and middle-income countries is often subject to stifling resource, time, and administrative constraints—especially in the case of small-scale and Ph.D. student research projects. A typical challenge in this respect is the absence of sampling frames for rural and urban household surveys in low- and middle-income countries, for example in cluster random sampling designs [1, 2].

Survey researchers conventionally rely on manual household listing and mapping approaches to address the problem of missing sampling frames, but this approach comes with high resource requirements. In order to manage costs and workload, often only segments of larger clusters are listed, or researchers rely on random walk approaches without constructing a sampling frame [3]. Such economising solutions can result in a clustering of responses if nearby dwelling units share similar characteristics (e.g., because they are located in a slum area). Because it is difficult to locate clearly the boundaries of a cluster (or village) in these approaches, it is also possible that marginalised households at the village fringes are more likely to be omitted from the sample than centrally located dwelling units.

The increasing availability of online maps, satellite imagery, and digital technology can facilitate the sampling process in low- and middle-income contexts. For example, Wampler et al. [4] carried out cluster random sampling in Haiti and Escamilla et al. [5] implemented a simple random sampling design in Malawi. In both cases, the authors use Google Earth to map the sites and mark eligible structures for sampling, geographical information system (GIS) software to extract the coordinates of the identified structures, additional software tools to select the sample based on the list of coordinates, and global positioning system (GPS) handhelds to upload the sample coordinates and to locate the houses in the field. Besides, Escamilla et al. [5] use GPS handhelds to define the survey site from which they draw the sample.

A slightly different solution is proposed by Shannon et al. [3]. Although the authors use Google Earth as well, they also utilise commercially procured aerial maps. From these geo-coded sources, Shannon et al. [3] sampled coordinates randomly and selected housing structures within a 20 m radius of each location. As the number of structures in each location was known through manual enumeration, the authors applied sample weights to each selected building to improve the socio-geographic representativeness of the sample. In addition, rather than uploading the sample coordinates to GPS handhelds, survey fieldworkers were equipped with printed maps to locate the households.

Satellite-aided approaches are also employed to facilitate random walk strategies. Galway et al. [6] describe a technique in which they export geospatial cluster data from GIS software to Google Earth, subsequently superimpose a grid and randomly choose cells therein as starting points for random walks in their study site. Like Shannon et al. [3], Galway et al. [6] face a security-sensitive research environment and use printed maps rather than handheld GPS units to locate the starting household in the field. Not relying on any specialised software or data source, Flynn et al. [7] use the browser-based Google Maps service to randomly select the starting point for random walks in urban environments in Canada. Moreover, satellite-aided approaches can also be found in programme monitoring and disease surveillance, involving specialised GIS software in nearly all cases [8–10].

These new satellite-aided survey sampling and implementation approaches face a number of difficulties. One problem is the reliance on specialised software and user skills, professional GPS equipment, and/or commercial data sources. This can make the strategies inaccessible or unattractive to researchers from a methodological and financial standpoint. The only study where no specialised skills, equipment, or data are required is Flynn et al. [7], where, however, the sampling strategy is limited to a random walk. Furthermore, despite utilising satellite imagery and aerial maps, spatial considerations in the sampling strategy mostly focus on defining the catchment area. The aforementioned solutions are useful to ensure the inclusion of marginalised houses in the sampling frame, but they do not automatically lead to improved spatial representation. The sub-cluster weighting approach of Shannon et al. [3] is a notable exception, yet also this solution does not guarantee that the sample is spatially stratified. An additional problem is to locate selected houses using GPS units, which can make it difficult to distinguish neighbouring dwelling units. In some situations, the conspicuous use of GPS handhelds can also pose a security issue.

The objective of this paper is to discuss the advantages and limitations of an alternative approach to satellite-aided survey sampling that (a) can make survey sampling and implementation feasible where conventional approaches would be logistically challenging and prohibitively expensive; that (b) does not require specialised software skills, equipment, or data sources; and that (c) enables better spatial representation of rural communities in low- and middle-income contexts. To this end, I describe two household surveys in rural Rajasthan (India) and rural Gansu (China) and compare the sampling accuracy and costs between conventional household listing and my satellite-aided approach.

In contrast to other strategies utilising satellite imagery, the procedure presented in this paper does not require any specialised software knowledge nor professional equipment to locate households. Latest yet basic off-the-shelf laptop computers, low-cost smartphones, printed maps, and browser-based map services such as Google, Bing, and HERE maps are sufficient to select the sample and to facilitate implementation [11–13]. In addition, by ensuring better spatial representation of the survey areas, the procedure presented here is likely more efficient than conventional cluster sampling approaches with random walk procedures [6, 14, 15]. The approach proposed in this paper is therefore intended as an addition to survey researchers’ methodological toolbox.

## Methods^1^

I illustrate my low-cost/low-tech approach through a case study analysis of two surveys in rural Rajasthan (India) and rural Gansu (China). In Rajasthan, administrative, logistical, and economic conditions permitted conventional survey sampling and implementation. The survey context was more constrained in Gansu, where high labour costs, logistical challenges, and administrative constraints rendered the conventional approach economically infeasible.

I describe the survey case studies to highlight that the tools and techniques involved in my new approach can replace conventional survey sampling approaches, and that they require a lower level of technical sophistication than existing satellite-aided approaches. In addition, the Gansu case study outlines opportunities to appreciate spatial considerations in survey sampling that go beyond conventional and other satellite-aided approaches.

In order to assess the suitability of my approach, I describe the survey results from Rajasthan and Gansu in terms of identification accuracy and refusal numbers, considering that satellite maps allow the researcher to identify houses but not necessarily households. I also compare the financial costs and benefits of my survey sampling approach with the expected costs of conventional household sampling across various scenarios.

## Results

### Survey case studies

The surveys described in this section are part of a mixed methods study on mobile phone diffusion and healthcare access in India and China. This research was carried out among the adult village population in selected districts of Rajasthan (Udaipur, Rajsamand) and Gansu (Baiyin, Dingxi, Lanzhou). Following a preceding qualitative research stage, the field sites were revisited in order to test hypotheses derived from a qualitatively grounded theoretical framework.

No prior study had been conducted on the studied phenomena (i.e., the effects of phone diffusion on healthcare behaviour). Sample size decisions in this kind of exploratory research are difficult because commonly used statistical power calculations are not applicable for phenomena whose effect size is unknown a priori [16]. In addition, the survey had an explicit spatial component because my analysis assumed that mobile phones influence people’s health behaviour differently depending on their location.

Given that this is the first study attempting to measure the effects of mobile phones on healthcare access on the micro level, sample size considerations focused on assumptions of the prevalence about phone-aided health action in the population based on the preceding qualitative research. Ideally, this would have involved a simple random sample across my field sites with sample sizes in excess of 500 per country in order to capture rare behaviours and to permit logistic regression analyses stratified by country. Resource constraints prevented such a survey design, instead requiring cluster random sampling in order to stay within the available budget by reducing survey fixed costs [17]. At the same time, spatial sample stratification helped to improve the effective sample size, which would otherwise be diminished by cluster random sampling designs [18]. The multi-stage stratified cluster random sample results directly from these considerations and constraints. The sample size was limited to 400 respondents per site because of budget limitations (approx. £11,000 per country).

The survey design therefore followed a four-step process in order to survey 400 rural dwellers in each country:

1. purposive selection of representative sub-districts in each study area;
2. random selection of 16 villages across these sub-districts per country;
3. random selection of 25 households in each village through interval sampling;^2^ and
4. random selection of one respondent in each household through the use of age-order tables [19].

#### Rajasthan: village and household sampling

The first survey was implemented in Rajasthan, with assistance from the Indian Institute of Health Management Research (IIHMR), Jaipur. I keep the description of village and household sampling short because very few difficulties were encountered and the survey therefore adhered to standard practice.

Village selection in Rajasthan took place without notable complications. Village-level census data was available and Valid International provided geographical location data for all registered villages in Rajasthan. I sampled the villages proportionally to population size and stratified by their distance to the nearest sub-district town (above or below sub-district average distance). I used the geographic coordinates to calculate these distances. Two villages of the original sample had to be replaced because heavy rainfall made them inaccessible.

Following the village sampling, a team of six field investigators and two supervisors relocated to the survey sites. Half a team day each was required to map the village and list all households in the selected areas (i.e., two segments of less than 250 households in villages larger than 500 households). Village segmentation was required in order to maintain a manageable workload. This was the case in 6 of the 16 villages, in each of which the number of segments ranged from three to six. These segments covered on average 55 % of the total number of households per village. Overall, the estimated population in the total or partial villages ranged from 161 to 1272.

After the household listing, one team-day per village was required to approach, interview, and, if necessary, revisit 25 households in each village. An interval sample was drawn from this household list based on the total number of households in the segment and a randomly selected starting point, accounting for up to ten replacement households (i.e., 40 % oversampling).^3^

#### Gansu

Contrary to expectations, the sampling strategy could not be replicated in Gansu. Neither village-level census information nor geographical data was available—merely a register containing village and township names could be accessed [21]. In addition, financial resources were insufficient to list households in the villages through the local survey team. Despite the administrative and resource challenges, the Gansu survey would have to be implemented in a comparable four-stage design as in Rajasthan. I describe in the following how satellite mapping solutions and other digital aides compensated for the lack of administrative data and facilitated sample selection and survey implementation.

##### Village and household sampling

The village register was the only available administrative data source for the village selection, but it did not contain population or geographical data. While population data on the registered villages remained inaccessible, a data set containing geographical village coordinates could be constructed using Google Maps (this could have been done alternatively with the free software Google Earth; Bing Maps and HERE Maps proved inferior to Google Maps for locating Chinese villages [11–13, 22]).^4^ I located the registered villages through Google Maps manually, using their Chinese names. The process was repeated for 1736 registered townships and villages in the eight selected sub-districts in Gansu. This procedure did not only help to extract the village coordinates, but it also served to validate the information in the village register. A number of entries had to be removed from the list because they were urban areas, duplicates, or because they could not be located in Google Maps or elsewhere.

When the data extraction was complete, I calculated the distance between each village and their associated townships. While the main purpose of the distance calculation was village sample stratification, it also helped to detect outlier villages with extreme distances to the township, which led these villages to be re-entered, re-classified to another township or district, or dropped. After validating the geographical data, 1553 villages remained in the sampling frame.

I selected 16 villages plus 32 replacement villages in Gansu, which tended to be larger than their Rajasthan counterparts, ranging from estimated 481 to 3141 inhabitants (estimates based on survey data).^5^ Two villages had to be replaced because weather conditions rendered them inaccessible; one village was replaced because it did not have high-resolution satellite imagery available.

Following the village selection, the online map and satellite image providers Google Maps and Bing Maps facilitated the selection of survey households (in many instances, Bing Maps provided superior-quality images). Given that resource limitations were an obstacle for household listing through the survey teams, I relied on satellite imagery to replace this process. All selected villages or their first replacement had high-resolution satellite images available on Google Maps, Bing Maps, or both (the highest available resolution was 1:670).

**Fig. 1:**
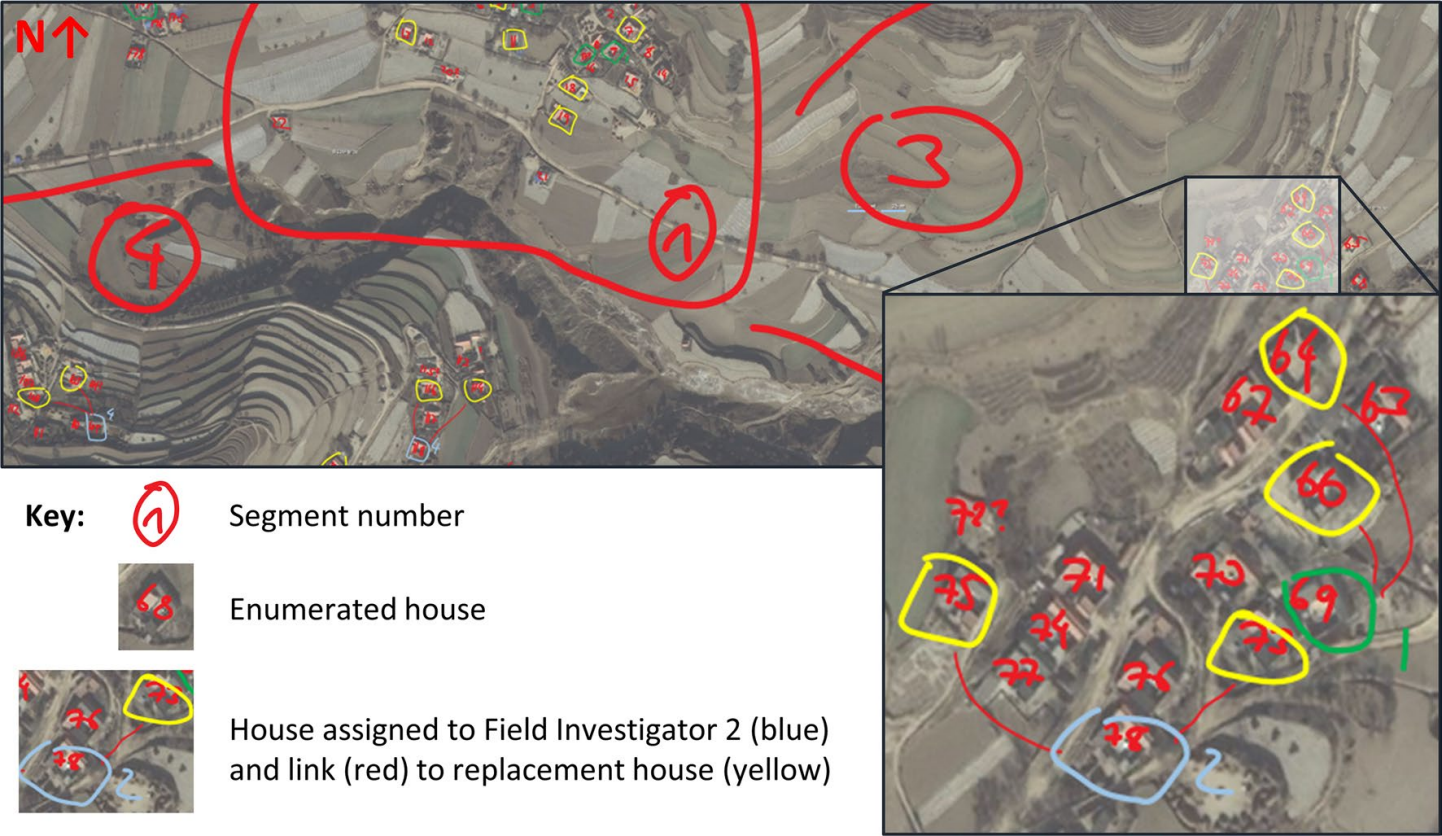
Excerpt of segmented and numbered village map with detail. Source: Survey material for survey fieldworkers, 2014 map data from Microsoft Corporation, DigitalGlobe, HERE

In order to extract the satellite maps, a catchment area within a 1 km radius from the selected village centre was screen-capped at the highest resolution (uninhabited areas omitted), pasted into Microsoft PowerPoint, and re-assembled to yield one high-resolution map of the catchment area (see Fig. 1 for an extract; the detail shows the numbered houses, the selected structures, and the assigned field investigators as explained in the following paragraphs). This map was further complemented with lower-resolution overview maps that indicated approach roads and nearby towns for navigation. A complete area map of a village could thus comprise between 5 and 40 individual high-resolution maps, containing up to 950 residential buildings or 380 on average per village.

Once the complete location map was assembled, I divided the village (or settlements) into natural segments to ensure that both central and marginal areas were represented in my survey. For example, if a village comprised one core segment of 400 houses and an adjacent yet detached group of ten residential buildings belonging to the village, the latter group would form a separate segment with at least one sampled household. Segmentation was necessary for this survey because random sampling would not guarantee that households at the village margins are included in the sample.

The next step in the process consisted of identifying and listing all residential properties in the village. Housing structures in the field site were homogeneous, consisting of yards surrounded by two or three buildings and encircled with a wall and a gate. None of the villages contained apartment buildings. The detailed aerial images therefore enabled reliable identification and consecutive numbering of houses using the annotation features of Microsoft PowerPoint and a touchscreen laptop (i.e., each residential structure was labelled with a handwritten number; see detail in Fig. 1).^6^ Difficulties only arose with respect to other village structures that were later identified as schools, village councils, or factory buildings. These structures received placeholders to be included in the sampling frame should they prove to be residential during the village visit. This was not the case, however.

In accordance with the Rajasthan leg of the study, I selected the household sample through systematic random sampling based on the household number per segment, a random starting point, and a fixed interval [23]. Interval sampling was chosen to ensure a better spatial representation of the segment than a simple random draw.

I selected 75 houses per village through this method, which includes 50 replacements or two for each selected house because it is not clear beforehand whether these buildings are locked or abandoned. The replacements—marked in yellow in Fig. 1—were located within the intervals (i.e., an interval was selected for 75 houses, and every third house is the first choice, followed by two replacements). The sample size per segment was assigned according to the number of residential structures.

This process allowed the mapping and listing of all residential structures in one village by a single person within 3–5 h [compare this to the approx. 32 labour hours required to list all households in a village in the Rajasthan survey (including investigators, supervisor, and driver)]. As this sampling strategy could be executed without actually visiting the villages, and because neither salary nor additional equipment were required for the lead researcher (a Ph.D. student), the process took place without notable costs.

While I chose interval sampling stratified by village segments, other survey designs can superimpose a grid structure on the villages to select households [6, 24]. Where spatial considerations are not relevant for sample selection, researchers can consider random draws from the sampling frame, which can be created through map annotations or through Google Earth as in Wampler et al. [4] and Escamilla et al. [5].

##### Survey implementation

Digital technology did not only facilitate village and household selection, but it also simplified the field investigators’ and supervisors’ work, and it helped to locate and approach the villages and households.

**Fig. 2:**
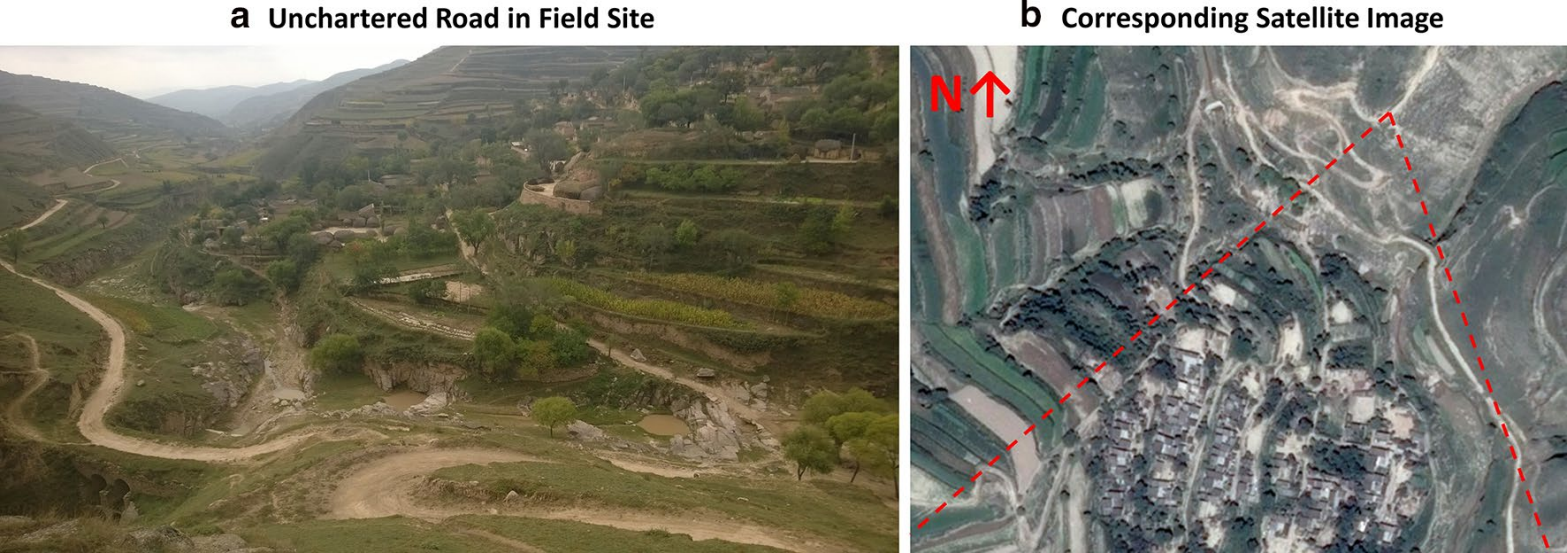
Example of unchartered road and corresponding satellite map. Source: own photograph from field survey; 2014 map data from Google Inc., CNES/Astrium. *Dotted red lines* in **b** indicating perspective of photographer

The detailed satellite maps enabled up-front planning of the route to each village because—unlike road maps—major and minor roads can be easily discerned even if they are not officially mapped. When approaching the village, a low-budget smartphone with satellite map applications (Lumia 638 with HERE Maps) enabled the team to precisely track the current position of the vehicle and to select the correct approach road (see Fig. 2 for an example of unchartered approach roads and their representation on a satellite image). This saved valuable time, which the team could then spend more usefully on carrying out the survey.

I used the numbered village maps to assign each of the six field investigators to their survey households, to plan the drop-off of the investigators in different segments of the village, and to coordinate the final meeting and pick-up of the investigators prior to departure. This level of planning was necessary in order to complete 25 1-h survey questionnaires during 1 day, considering travel times of up to 3 h to the village.

The detail in Fig. 1 illustrates the investigator assignment through numbered boxes drawn around the selected houses and their replacements (in this case, PowerPoint’s annotation features are superior to markers in Google Earth). The selected houses were assigned to the investigators in such a way that relocation time and distance between the interviews was minimal. In addition, because the maps make clear from the very beginning which segments are relatively difficult to access, more athletic investigators could be assigned to more demanding areas. All these planning activities helped to reduce slack time when approaching and navigating the villages, making the village visit as efficient as possible.

In order to facilitate the work of the field investigators, each investigator received a complete set of A4 print copies of the village maps. Reducing an entire area map to the size of A4 for the investigators is impractical for household selection because maps details become indistinguishable. Handling maps larger than A4 is similarly cumbersome for the field investigators (e.g., poster-sized at highest resolution). I instead extracted and magnified the map segments relevant to each investigator, and provided lower-resolution overview maps to facilitate village navigation. While the resolution of the detailed village maps varied, these variations were of no consequence for the survey implementation because the field investigators received the maps in advance and could raise concerns if they were unable to identify their assigned houses.

The survey supervisors briefed and accompanied the investigators where they were unsure about the exact location of a house (e.g., in the centre of a densely populated village). A compass, their own smartphones with satellite navigation applications, and local residents further helped the investigators to navigate the village and approach the correct household according to their maps.

### Sampling accuracy

The final samples in Rajasthan and Gansu comprised 400 adults each. In Rajasthan, 33 replacements (or up to eight in one village) were necessary because the selected respondents were unavailable or refused participation. In Gansu, the satellite-based approach did not permit judgements as to whether the selected houses were inhabited or vacant. A total of 223 houses or up to 29 houses per village had to be replaced because they were vacant or locked. Manual household listing and mapping through the survey team—had it been possible—would have filtered out a large portion of this number by design. In addition, refusal was slightly higher in Gansu as well, with a total number of 38 households, or up to four in a single village. The surveys therefore required 33 replacements in Rajasthan and 261 in Gansu.^7^

Whereas houses in Gansu were often found vacant, it was rarely the case that houses were omitted from the sampling frame or inhabited by more than one household. Only one house was shared by two households, and initially unidentified structures turned out to be schools, factories, and village councils. A notable mismatch between the satellite maps and the village realities arose only in one village where an entire segment of buildings was still under construction and thus uninhabited. “On-the-fly” updates of the village samples using the printed maps and a recalculated sampling interval helped to solve this problem prior to commencing the village survey.

### Financial cost-benefit scenario analysis

Using satellite imagery through online map services and other digital aides enabled low-cost survey sampling and streamlined implementation logistics. This relaxed otherwise binding constraints of available team time and had direct financial implications. But not all of the cost savings and efficiency improvements are quantifiable. For example, navigating unchartered rural roads with satellite maps saved travel time (compared to using road maps and asking for directions). While the time savings were beneficial for team performance and morale, their financial impact was limited because team members were paid on a per diem basis.

In order to provide an indication of the relative financial costs and benefits involved in my satellite-aided sampling strategy, I compare in Table 1 the savings and expenditures involved my chosen strategy in Gansu versus three scenarios involving conventional household listing. The three scenarios are based on actual field experiences: Firstly, the optimistic scenario assumes that the team can completely list two villages in 1 day, with limited overnight stays required. The second, more realistic, scenario appreciates the dispersion of the villages in rural Gansu, making it more difficult to access and map two villages in 1 day. In a third and yet more conservative scenario, it would only be possible to map and list one village per day, requiring up to eight overnight stays for the team.

**Table 1:** Cost comparison of conventional versus actual sampling strategy in Gansu

Assumptions	Scenarios Conventional household listing			Actual satellite-aided household listing		
	Optimistic (easy access)	Realistic (medium access)	Conservative (difficult access)			
Number of sites for listing	16	16	16	16		
Number of team days per site	0.5	0.75	1	0		
Number of required overnight stays	4	6	8	0		

Household listing costs in planned design			Optimistic	Realistic	Conservative	Actual
Item	Unit	Costs/unit	Total costs	Total costs	Total costs	Total costs
Field investigator (×6)	Daily rate p.p.	£18.42	£884.21	£1326.32	£1768.42	
Supervisor (×2)	Daily rate p.p.	£23.68	£378.95	£568.42	£757.89	
Vehicle (incl. driver, fuel)	Daily rate	£76.84	£614.74	£922.11	£1229.47	
Insurance (×8)	Daily rate p.p.	£0.17	£11.02	£16.53	£22.05	
Team accommodation (×9)	Night p.p.	£15.79	£568.42	£852.63	£1136.84	
Overseas travel allowance	Daily rate	£50.00	£400.00	£600.00	£800.00	
Smartphone	Unit	£100.00				£100.00
Printing	Total charges	£40.00				£40.00
Mobile data charges	Total charges	£30.00				£30.00
Labour costs for satellite sampling	Hourly rate	£15.00				(£960.00)
Touchscreen laptop	Unit	£700.00				(£700.00)
GPS units	Unit	£18.00				(£180.00)
Total			£2857.34	£4286.01	£5714.68	£170–2010

Source: own elaboration

Data based on actual expenditures. Assumed exchange rate: GBP 1.00 = CNY 9.50. Expenditures in parentheses are likely to arise in other research but did not accrue in the present study

Each of these scenarios depicts the estimated costs of the household listing process, including daily allowances, transportation, insurance, and accommodation. Also an international travel allowance of £50 per day is calculated, given that the conventional strategy would effectively extend the total duration of the survey. The estimated household listing costs thus range from £2860 to £5710, depending on the difficulty of village access.

In contrast, the actual expenditures in this survey pertain to mobile phone equipment and operating costs to implement the strategy. These expenses amounted to approximately £170. In other circumstances, researchers may have to budget their own work time spent on mapping the households and acquire an adequate laptop computer and GPS units to cross-check the field investigators’ household selection. These optional expenditures are indicated in parentheses and amount to £1840.

Compared to the assumed costs of conventional survey sampling in Gansu, my satellite-aided approach saved between £850 and £5540. In any of these scenarios, satellite-aided household selection proves cost efficient. Given total actual survey costs in Gansu of £11,470, the cost savings correspond to a 26 % reduction of the anticipated expenses of the Gansu leg of this study (given actual implementation costs versus the “realistic” conventional sampling scenario; if we include the “optional” costs, this would be a 14 % reduction). In addition, labour time savings for household sampling correspond to a reduction of the main survey time by at least 25 %.

The net benefit of this strategy depends on local cost conditions and the difficulty of creating the village sampling frame using conventional methods. In Rajasthan, the actual costs of conventional household listing were approximately £1250 (including staff, transport, and overheads), which is far below the potential expenditures for the satellite-aided approach (i.e., up to £2010). A satellite-aided survey sampling approach might not be required on financial grounds in such situations.

## Discussion

The survey sampling and implementation process outlined above had considerable advantages over conventional approaches, especially in financial terms. However, my proposed strategy is not universally applicable. Researchers intending to adopt a similar approach should be aware of the preconditions for utilising satellite maps and digital aides successfully, and of the remaining logistical and methodological challenges.

### Locational, technical, and logistical prerequisites

Survey researchers aiming to adopt the sampling procedure outlined in this paper have to be aware of locational, technical, and logistical factors that influence the viability and success of using satellite imagery and digital aides in their work.

Locational factors influence the feasibility and viability of a satellite-aided sampling strategy. First, if up-to-date high-resolution satellite images are not available via any provider (e.g., Google Maps, Bing Maps, HERE Maps), their use is evidently ineffective. The same would apply if housing structures are highly irregular, if they are indistinguishable from non-residential buildings, if the population is very mobile (e.g., nomads), or if rural dwellings generally accommodate more than one household, for example in the case of apartment buildings. Such conditions would hamper the correct identification of households, leading to the omission of parts of the population from the sampling frame. Knowledge of the local living conditions prior to the household sampling, for example through a qualitative pre-study, can help to detect such situations.

Locational factors also affect the economic viability of this sampling strategy. The strategy offers its benefits mainly in relation to missing household registers. Satellite-aided sampling approaches become less attractive if detailed administrative household lists are available (provided these lists are not politically influenced); where villages and households are easily accessible; where labour, transport, and accommodation costs are comparatively low; and if the survey focuses on a very small geographical area (e.g., one sub-district). The strategy is better suited to rural household surveys that extend over a large area and that involve dispersed settlements.

In terms of technical requirements, neither specialist equipment nor dedicated software packages are needed to implement this strategy (unlike e.g., [4–6]). I used off-the-shelf equipment and software (Lenovo Yoga, Microsoft Lumia 638, Microsoft Office 2013) to stratify the villages according to their distance to the nearest town, to label village households on the extracted satellite maps, to calculate the interval for household selection, and to locate and navigate within the selected villages. Laptop models with the required specifications (touchscreen, 8 GB RAM, 256 GB hard drive) currently trade at less than £500, Internet-enabled smartphones with an adequate satellite mapping application are available for less than £100. In general, it is possible to implement this strategy with basic and comparatively low-cost equipment, and without having to acquire specialised software skills.

Logistically, it is important that the lead researcher has at least basic training in survey sampling and is comfortable working with maps. In order to ensure the quality of the household sample, it is necessary to train the supervisors and field investigators intensively in using maps and to carry out briefing sessions in each village. Supervisors should be instructed in detail about the journey to the village and the deployment of the investigators on the day before the village is surveyed. In order to identify yet unknown structures in the village, it is also useful to discuss the locational specifics with village leaders before deploying the team.

### Challenges

Satellite-aided household sampling approaches come with an idiosyncratic set of advantages and challenges. Methodologically, map-based household selection makes it easier to list all households in a large village rather than only segments thereof, which can improve the representativeness of the sample [5]. In addition, the use of satellite maps enables spatial village stratification in order to ensure that marginalised dwellers are included in the sample as well (while I chose interval sampling stratified by village segments, other survey designs can superimpose a grid structure on the villages to select households [6, 24]).

As a special form of stratification, this approach is (in theory) at least as efficient as simple random sampling and superior to incompletely generated household lists in dispersed villages or approaches using a random walk (provided that observed effects are correlated across proximate households [6, 14, 15]). In other words, spatial sampling approaches can help to reduce the extent of clustering in a village, which can increase the effective sample size in complex multi-stage sampling designs [15, 18].

Despite these advantages, satellite-aided village and household sampling also raises methodological questions. One challenge arises from the use of Google Maps or Google Earth for listing villages and recording their coordinates. It is conceivable that road and satellite map information does not fully correspond to official village registers or that village registers are politically influenced, both of which can lead to the systematic exclusion of particularly small and remote communities. Though more time-consuming, an alternative to using village lists is to inspect the satellite imagery in the selected regions and record the location of all identifiable settlements.

In either case, compared to sampling through census data, the methods are insensitive to population size. Large and small villages are equally likely to enter the sample [2], which can bias the sample towards smaller villages. In order to correct for respondents’ higher probability of selection in smaller villages, the researcher can estimate the village population ex post based on the village household count (derived from the map-based household list) and the average number of household members in the surveyed dwellings. Sample weights can then correct for the higher chances of residents in small villages to be included in the sample [18].

Household sampling through satellite maps has at least two further methodological implications. Firstly, experiences from the Rajasthan leg of the study suggest that manual household listing through the survey team can be an opportunity to build trust with the residents before they are being surveyed. It is possible that this can reduce refusal rates compared to research teams who spend only 1 day in each survey village.

Secondly, even where housing units are homogeneous, it is difficult to identify shared and abandoned houses through aerial images. This is a disadvantage compared to manual household listing and mapping, which identifies households rather than houses and filters out uninhabited dwellings when establishing the sampling frame. If segments of the village contain a disproportionate share of vacant houses, then the inhabitants of this segment would be overrepresented in the village. It is therefore advisable to discuss the village maps with local leaders and update the sampling frame “on the fly” if necessary.

## Conclusions

This paper illustrated and discussed the merits, requirements, and challenges of using map services, satellite imagery, and basic IT equipment to facilitate sample selection and survey implementation in a rural low-income context. I argued that, despite its challenges, this strategy can be a cost-efficient and transparent alternative to conventional village and household listing methods. The cost-benefit scenario analysis underlined the usefulness of my approach in the Gansu context with high economic and logistical constraints, whereas a conventional approach can be preferable in the Rajasthan setting with low survey implementation costs and few administrative constraints.

Provided minimal investments in basic equipment, this approach can be replicated in other contexts where resources for household listing are limited, where sampling frames cannot be produced from administrative data, and where residential structures are homogenous and distinctive. Satellite-aided survey sampling and implementation helped to reduce main survey time by one quarter and saved approximately £4100 in the present study. This and similar approaches can therefore potentially improve the affordability of surveys especially for student researchers and resource-constrained studies.

## Abbreviations

2G: second generation
CNY: Chinese Renminbi Yuan
GB: gigabyte
GBP: pound sterling
GIS: geographical information system
GPS: global positioning system
IIHMR: Indian Institute of Health Management Research
IT: information technology
RAM: random access memory
VPN: virtual private network

## Footnotes

1 **The research was approved by the Oxford Department of International Development’s Departmental Research Ethics Committee in accordance with the procedures laid down by the University of Oxford for ethics approval of all research involving human participants (CUREC1A/ODID C1A 14-031).**

2 I defined a household as a group of people who normally live in the same dwelling, eat together, who may or may not be related, and who had resided in the village for at least 6 months prior to the survey.

3 The survey team administered paper questionnaires, which were subsequently entered into the CSPro software [20].

4 In contrast to Google Maps, Google Earth would enable exporting the coordinates in the metric Universal Transverse Mercator coordinate system, which does not require the researcher to apply trigonometric formulae in order to calculate distances. Whether it is more time-saving to manually extract the coordinates from a browser-based interface or to automatically export the coordinates of pinned locations in Google Earth ultimately depends on the researcher’s skills and preferences. I thank the anonymous referee for pointing this out.

5 Part of the reason for the larger village size (apart from socioeconomic differences between the sites) is the 1 km catchment area in Gansu, meaning that surrounding settlements could be included in the sample. A primary sampling unit therefore does not necessarily correspond to one village.

6 As an alternative to manual labelling, researchers using Google Earth could mark and export all residential structures (this would forgo the option of using alternative satellite map services such as Bing Maps).

7 After the data were entered, first-stage error rates were 0.074 % per data field in Rajasthan and 0.035 % in Gansu. All these issues were clarified or rectified after revision.
